# Graphene-Based Chemical Field-Effect Transistors: Impact of Electric Double Layer Model and Quantum Capacitance on Na^+^ Detection Capabilities

**DOI:** 10.3390/mi17040433

**Published:** 2026-03-31

**Authors:** Ghassem Baridi, Arslan Liaquat, Leonardo Martini, Luca Nappi, Federico Rapuzzi, Vito Clericò, El Hadj Abidi, Yahya Moubarak Meziani, Mario Amado, Enrique Diez, Giorgia Brancolini, Luigi Rovati, Francesco Rossella

**Affiliations:** 1 Department of Engineering “Enzo Ferrari”, University of Modena and Reggio Emilia, Via P. Vivarelli, 10, 41125 Modena, Italy; luigi.rovati@unimore.it; 2 Department of Physics, Computer Science and Mathematics, University of Modena e Reggio Emilia, Via Campi 213/a, 41125 Modena, Italyf.rossella@unimore.it (F.R.); 3 Department of Applied Physics, University of Salamanca, 37008 Salamanca, Spain; 4 Istituto Nanoscienze—CNR, S3, Via G. Campi 213/A, 41125 Modena, Italy

**Keywords:** graphene field effect transistor, electric double layer, quantum capacitance, biosensors, electrolyte gating

## Abstract

Graphene-based ion-sensitive field-effect transistors can operate as biosensors by utilizing the formation of an electric double layer at the interface between the electrolyte and the graphene channel, enabling high sensitivity, scalability, and cost-effective fabrication. In this work, we focus on the working principles and current methodologies associated with these devices, making a comparative analysis of different models that describe the electric double layer in the electrolyte, referring to sodium ions (Na^+^) as a case study for the detection performance of the graphene biosensor, and taking into account the impact of graphene quantum capacitance. Our study addresses the sensitivity of graphene field-effect transistors within the framework of the Gouy–Chapman model, as well as the Stern model, computing device sensitivities of 3200 V/M and 5500 V/M, respectively. By incorporating the impact of graphene’s quantum capacitance in the calculations, increased sensitivity up to 5620 V/M was found. The present work shines light on the rationalization of graphene-based biosensors’ operation and performance.

## 1. Introduction

Sensors, detecting changes in physical, chemical, or biological parameters of the environment and transforming them into measurable signals [1], are commonly applied in multiple application areas, such as medical diagnostics, food quality assessment, industrial process regulation, and environmental surveillance, to name a few [2,3,4,5]. A quite peculiar family of sensing devices is represented by biosensors, which typically envision the synergic operation of three different systems: a bioreceptor to bind the target analyte, a transducer to convert a physical or chemical change into a measurable signal (e.g., an electric signal), and a processor to read the signal and display the output [6,7,8,9].

Nanostructured materials are widely used for electrical biosensing applications because they allow enhancement of the stability of bioreceptor attachment on the electrode surface by enabling a more electroactive interface through functional groups [10]. Furthermore, the use of nanomaterials can promote the enhancement of molecular adsorption, the acceleration of signal response, and the improvement of the stability of the electrodes. In addition, the high surface area displayed by most nanomaterials usually boosts enhanced sensitivity, catalytic activity, broad dynamic range, operation at low potential, and efficient electron transfer from the electrode’s active site, making nanomaterials very good candidates for the development of efficient electrochemical nano-biosensors [11].

Among the different families of nanomaterials, carbon-based ones stand out for their excellent electrocatalytic activity and electrical conductivity. While these materials come in various forms, graphene has attracted particular attention due to its exceptional electrical properties, such as high carrier mobility and conductivity, rapidly becoming an extremely promising system for the use in nanoelectronic devices including biosensors [12,13,14,15,16], where it emerged as a unique sensing element [17,18] combining flexible structure, large surface area, and exceptional electrical, mechanical, and optical properties. Moreover, as graphene is a zero-bandgap material (or semi-metal), in graphene-based devices, the charge carriers within the 2D channel can very easily vary from electrons to holes through the application of an electrostatic gate, with the transition occurring at the minimum charge carrier density, a condition referred to as the Dirac point [18,19]. Notably, this makes graphene extremely responsive to external perturbations, such as electric fields, chemical doping, and mechanical deformation [20,21]. In addition, based on the unique atomic thin-layer structure, the electrical properties of graphene are extremely sensitive to the presence of external atoms or absorbed molecules [12].

Computational and theoretical approaches have proven valuable for designing nanomaterial-based sensors by enabling the prediction and optimization of molecular interactions at the nanoscale. Among these materials, graphene has been extensively explored as a sensing platform due to its tunable electronic and surface properties [22]. Graphene field-effect transistors (G-FETs) provide one of the most successful platforms for the detection of biological species. These devices typically envision a graphene channel—electrically contacted by source and drain electrodes—with lengths and widths of up to several tens of micrometers, and a thickness of one atomic layer (typically assumed to be 0.33 nm) lying on a dielectric film (e.g., ${SiO}_{2}$) that is typically 100 s nm-thick, grown onto a highly doped bulk substrate (e.g., Si^++^). They are three-terminal electronic devices, where the current flow between source and drain electrodes is modulated by the electric field induced at the dielectric surface when gate voltage is applied to the substrate [23,24]. Importantly, in G-FETs, the electrical transport properties of the graphene channel might change very finely upon the occurrence of chemical doping as well as electrostatic gating, including electrolyte gating [24,25,26,27]. At high carrier densities, such modulation is strongly influenced by graphene’s quantum capacitance, as demonstrated experimentally in ion-gated graphene devices by Ye and co-workers [28]. The relevance of quantum capacitance to sensing performance has also been evidenced in other 2D-material systems, such as MoS_2_-based field-effect biosensors [29]. Electrolyte gating exploits mobile ions in liquid electrolytes to build up an electric double layer (EDL) at the interface between the electrolyte and the graphene channel, generating a strong interfacial electric field that modulates the electrical current in the device. When changes occur in the electrolyte environment, the graphene Fermi level is shifted, inducing a shift in the Dirac point, which is probed by the response curve (transconductance) of the electrolyte-gated G-FET [28,30].

The theoretical results are in qualitative agreement with the experimental observations reported by Y. Sofue et al., who observed a shift in the Dirac point in graphene in the presence of Na^+^ ions. In that experiment, the Na^+^ concentration was very low, whereas the theoretical model considers a higher ion concentration. This difference in concentration mainly affects the magnitude of the Dirac point shift, while the underlying physical mechanism remains unchanged. Therefore, these results can be regarded as an extension of the experimental findings to a higher concentration regime [31].

At the microscopic level, the formation of the EDL and the associated interfacial charge storage have been investigated through first-principles simulations, offering direct insight into ion–electrode interactions and capacitance at electrified interfaces [31]. Complementary molecular simulations using constant-potential methods have further clarified the structure and dynamics of ionic liquid/graphene EDLs, highlighting the critical role of electrode polarization in capacitive charge storage [32].

Recent studies have significantly advanced the understanding of electric double-layer (EDL) phenomena and charge transport in low-dimensional and hybrid material systems. Eisensmith et al. examined the critical conditions required to observe cross-quantum capacitance effects in electric-double-layer-gated transistors based on two-dimensional materials, emphasizing the influence of device architecture and measurement protocols. Francis et al. investigated charge-transfer mechanisms in graphene–quantum dot hybrid systems, demonstrating their potential for high-sensitivity biosensing applications. In a related context, Tene, Talia et al. systematically studied the roles of graphene oxide and reduced graphene oxide in electric double-layer capacitors, providing key insights into their electrochemical behavior and interfacial charge storage mechanisms. Furthermore, Mah et al. reported enhanced synaptic performance in neuromorphic devices through the synergistic interplay of indium tungsten oxide-based electric double-layer effects and electrochemical doping processes [33,34,35,36].

In the proposed model, the electrolyte is modeled as an ideal symmetric monovalent system, and the interaction between the electrolyte and the graphene channel is treated purely within an electrostatic framework. Effects such as specific ion adsorption, ion hydration, and detailed surface chemistry or functionalization of graphene are not explicitly included. These assumptions are made to isolate the fundamental electrostatic coupling governing the electrical double layer and the resulting Dirac point shift, while maintaining analytical and numerical tractability of the model. The present formulation is therefore expected to be valid in regimes where the electrolyte is dominated by monovalent ions, specific adsorption is weak, and deviations from ideal Poisson–Boltzmann behavior are minimal. In realistic biosensing environments, ion-specific effects, hydration layers, multivalent ions, and biomolecular adsorption may significantly modify the interfacial charge distribution and the electrical response of graphene. Incorporating such effects requires a more detailed treatment of ion–surface interactions and surface chemistry, which is beyond the scope of the present study and will be addressed in future work.

To the best of our knowledge, previous theoretical studies of electrolyte-gated graphene devices neglect or oversimplify the role of graphene quantum capacitance by treating it independently of the electrolyte double layer [37,38,39]. The present study incorporates quantum capacitance self-consistently within the electrostatic model: this is essential for accurately capturing the Dirac point shift and sensitivity, particularly near charge neutrality, and allows a quantitative assessment of its interplay with electrolyte screening effects.

The present model simulates the spatial distribution of monovalent ions (Na^+^ and Cl^−^) in a NaCl solution near a negatively charged graphene surface. Electrostatic interactions cause Na^+^ ions to accumulate near the interface, while Cl^−^ ions are repelled, leading to the observed potential changes and Dirac point shift. It should be emphasized that the model does not provide experimental detection of Na^+^ ions as an ion-selective electrode would; instead, it describes the redistribution of ions due to electrostatic effects.

In this work, we computationally investigate sodium (Na^+^) ion-based electrolyte-gated GFETs, resorting to the finite element method, exploring the impact of the Na^+^ concentration on the electrical transport properties of the devices. We report the results of the comparative analysis of different models describing the EDL in the electrolyte, assessing the detection performance of the graphene sensor, and considering the impact of graphene quantum capacitance. Device sensitivity is computed in the frame of the Gouy–Chapman model and the Stern model, obtaining values of 3200 V/M and 5500 V/M, respectively. By incorporating the impact of graphene’s quantum capacitance in the calculations, increased sensitivity up to 5620 V/M was found. Our work shines light on the rationalization of graphene-based sensors’ operation and performance and might boost the development of more efficient biosensors relying on the unique electrical transport properties of carbon-based layered materials and other families of 2D materials.

## 2. Theory

**Figure 1:**
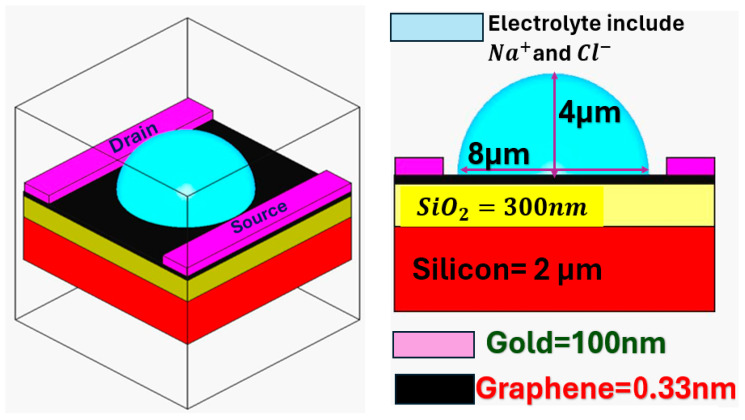
(**left**) Schematic diagram of a graphene field-effect transistor (GFET) gated by an electrolyte. (**right**) Cross-sectional view of the device. Red, yellow, black, light-blue, and violet colors are used for the silicon substrate, the silicon oxide dielectric, the graphene layer, the electrolyte with Na^+^ and Cl^−^ ions, and the metallic electrodes, respectively.

The G-FET architecture used in the present work is depicted in Figure 1. In the left panel, the three-dimensional rendering of the GFET is reported, showing the graphene channel (gray-colored) between source and drain electrodes (yellow colored), the dielectric film (red), the bulk substrate (black), and the droplet of Na^+^ ions-rich electrolyte (purple). The right panel reports the cross-sectional view of the device, indicating the dimensions of each device component (graphene channel, source/drain electrodes, overall substrate, and electrolyte droplet).

Device operation stands on the onset of the EDL at the electrolyte–graphene interface: a layer of Na^+^ ions accumulates in the electrolyte close to the graphene channel, while electrons accumulate in graphene. The EDL plays a crucial role also in electrochemical systems, as it might be the site of electrochemical reactions [32,40], and can be rationalized by models of increasing complexity, including the Helmholtz model, the Gouy–Chapman model, and the Stern model [41]. While these models are reviewed in detail in the Supplementary Materials, a summary of the main aspects and assumptions of the models is reported as follows.

The Helmholtz model was the earliest, most straightforward one for outlining the onset and the structure of the electric double layer (EDL). It assumes that the total charge in the electrode is counterbalanced by a single layer of oppositely charged ions in the electrolyte just at the electrode–electrolyte interface, forming the so-called Helmholtz layer of capacitance:

(1) $$C_{H}=\frac{\varepsilon_{0}\varepsilon_{H}}{d_{H}}$$

where ε_0_ is the vacuum permittivity, $\varepsilon_{H}$ is the relative permittivity of the Helmholtz layer, and $d_{H}$ represents the solvated ion radius (d/2). In this, the Helmholtz model overlooks the influence of the applied potential, the competition between the random thermal motion of ions and the directional forces due to the electrode’s polarity, and the interactions between the dipole moments of solvent molecules and the electrode surface. Since the electrolyte concentration plays a dominant role in determining the EDL capacitance over the concentration range relevant to our device, the assumption of a concentration-independent Helmholtz capacitance is not valid. Therefore, the Helmholtz model was not employed in our analysis, whereas models that explicitly account for concentration effects—such as the Gouy–Chapman or Gouy–Chapman–Stern frameworks—are more appropriate for describing the system studied in this work.

In the Gouy–Chapman model, the free charge refers to the electronic charge in excess that is responsible for current conduction in the graphene channel, which is balanced by an excess ionic charge distributed over a finite region of the electrolyte called the diffuse layer, where the electric potential decays gradually from the electrode.

In this framework, the differential capacitance per unit area reads:

(2) $$C_{Gc}=\frac{\varepsilon_{r}\varepsilon_{0}}{\lambda_{D}}cosh(\frac{2e\varphi }{2K_{B}T})$$

with Debye length $\lambda_{D}={\left(\frac{2ce^{2}}{\varepsilon_{r}\varepsilon_{0}K_{B}T}\right)}^{-\frac{1}{2}}\;$(for monovalent electrolyte), c as electrolyte concentration, e as electron charge, and $K_{B}T$ as thermal energy. The Stern model schematizes the electrochemical interface in two distinct regions: the compact or Helmotz layer, where the solution-side charge resides close to the electrode surface, and the diffuse region (corresponding to the Gouy–Chapman layer), where the remaining charge is spread throughout the solution, bounded by the compact layer and the bulk electrolyte. The EDL capacitance then reads [41,42,43,44,45,46,47,48,49]:

(3) $$\frac{1}{C_{EDL}}=\frac{1}{C_{H}}+\frac{1}{C_{Gc}}$$

In order to visualize the charge distribution and electrical potential on the surface of graphene induced by a gate voltage, we solve the Poisson equation, resorting to the semiconductor model available in COMSOL Multiphysics (Version 6.2) [50]:

(4) $$\nabla^{2}\varphi =\frac{-\rho (r)}{\varepsilon_{0}\varepsilon_{r}}$$

with ρ(r) as total charge density on the surface, φ as scalar potential, and relative permittivity [50]:

(5) $$\varepsilon_{r}=\frac{\varepsilon }{\varepsilon_{0}}$$

Sheet carrier concentrations (electrons or holes) in the source and drain regions can be approximated by [51]:

(6) $$n≅\sqrt{n_{0}^{2}+{\left(\frac{c_{back}\left(V_{backgate}-V_{dirac\;point}^{0}\right)}{e}\right)}^{2}}$$

where the back gate capacitance ($c_{back}$) is computed as [49,50]:

(7) $$C_{back}=\frac{\varepsilon_{r}\varepsilon_{0}}{t_{ox}}$$

where $t_{ox}$ is the thickness of ${SiO}_{2}$ (fixed at 300 nm).

The electrical current flowing through the graphene channel increases with the carrier density (*n*), which depends on how many target molecules are attached to the graphene surface [49,52]:

(8) $$I_{d}=\frac{W}{L}e*\mu *V_{DS}*n$$

Here, *W* and *L* represent the width and length of the graphene channel, respectively, *e* denotes the elementary charge, and *μ* is the carrier mobility. The source–drain voltage $V_{SD}\;$ is set to 1 mV in the calculations, if not specified differently.

When both a back gate and an electrolyte (top) gate are capacitively coupled to the same graphene channel (see Figure 1), the total parallel capacitance, expressed as specific capacitance (per unit area), is given by [53]:

(9) $$C_{total}=C_{back-gate}+C_{top-gate}$$

and the carrier concentration is modified in [52]:

(10) $$n≅\sqrt{n_{0}^{2}+{\left(\frac{c_{back}\left(V_{backgate}-V_{dirac\;point}^{0}\right)}{e}\right)}^{2}+{\left(\frac{c_{top}\left(V_{topgate}-V_{dirac\;point\;top}^{0}\right)}{e}\right)}^{2}}$$

where $c_{top}$ is the effective top-gate capacitance per unit area and $V_{dirac\;point\;(top)}^{0}$ is the Dirac point voltage due to the coupling of the back (top) gate.

Equation (10) is modified to the following form when back gate voltages might change while the electrolyte one does not [54,55]:

(11) $$n(V_{back-gate})≅\sqrt{n_{0}^{2}+{\left(\frac{c_{back}\left(V_{backgate}-V(x)-V_{0}\right)}{e}\right)}^{2}}$$

where V(x) is the potential along the source–drain direction, and $V_{0}\;$ corresponds to the Dirac point [54,55]:

(12) $$V_{0}=V_{back-gate}^{0}+\frac{C_{top}}{C_{back}}(V_{top-gate}^{0}-V_{top-gate})$$

## 3. Results and Discussion

**Figure 2:**
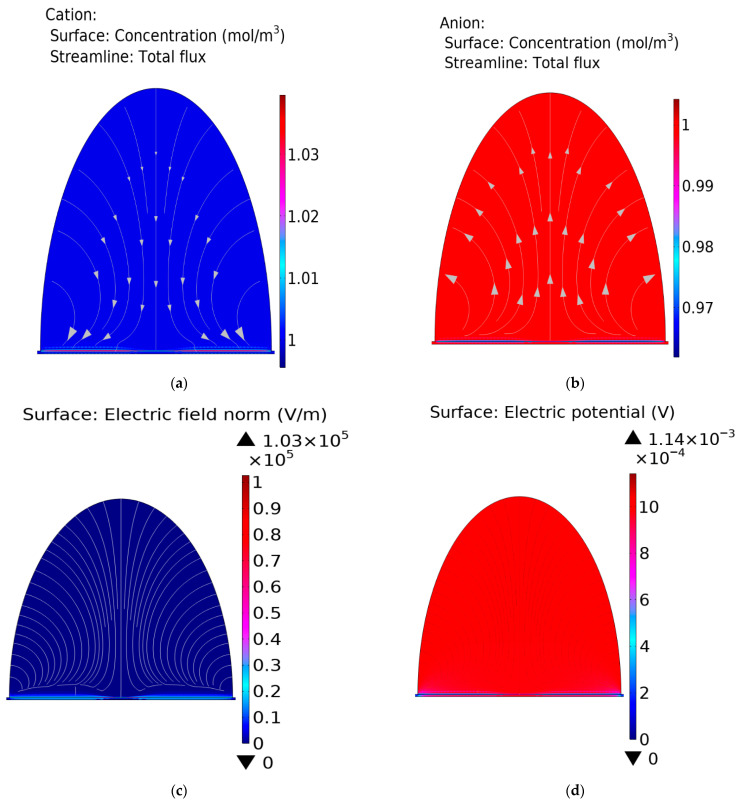
Panels (**a**–**d**) show the migration of cations (**a**) and anions (**b**), as well as the electric potential (**c**) and electric field (**d**) in the electrolyte, including the region near the electric double layer (EDL). The simulation uses the Stern model of EDL.

Using the device architecture illustrated in Figure 1, the key parameters associated with the different EDL models were evaluated. To this aim, we first simulated the migration of cations and anions within the EDL. For electric field and mass transport analysis, the electrostatic problem described by the Poisson equation, $\nabla \left(\varepsilon \varepsilon_{0}\nabla \varphi \right)=0$, was solved in the compact layer. In the diffuse layer, we used a coupled Nernst–Planck and electrostatic model [48,56] to capture the fine details of transport phenomena occurring in our device, calculating:

(13) $$\frac{{\partial c}_{i}}{\partial t}=\nabla (D_{i}\nabla c_{i}+z_{i}FD_{i}c_{i}\nabla \frac{\varphi }{RT})$$

with $\nabla \left(\varepsilon \varepsilon_{0}\nabla \varphi \right)=\rho$, where $\rho =\sum z_{i}c_{i}$ is solved in the electrolytic domain outside of the compact layer, and $D_{i}$, $z_{i}$ and $c_{i}$ represent the diffusivity, the charge valence, and the concentration of the ionic species *i*, respectively; *t* is the time, *F* is the Faraday constant, *R* is the gas constant, *T* is the absolute temperature, φ is the potential, ε is the dielectric constant, $\varepsilon_{0}$ is the vacuum permittivity, and *ρ* is the charge density. The results of the calculations are reported in Figure 2, which illustrates the migration of cations and anions, as well as the distribution of electric potential and electric field within the electric double layer (EDL), within the cross-sectional region of the device [48,56].

Cation and anion migration through the electrolyte are illustrated in Figure 2a and Figure 2b, respectively. White arrows in the Figures indicate the direction of ion movement: sodium ions (Na^+^), as cations, migrate toward the graphene surface, while chloride ions (Cl^−^), as anions, migrate away from the graphene surface. In both cases, small regions close to the graphene–electrolyte interface are zoomed in to highlight the occurrence of the EDL.

Electric potential and electric fields are reported in Figure 2c and Figure 2d, respectively. The EDL features are highlighted in the panels. When the electrode is polarized negatively with respect to the bulk solutions, it is negatively charged and attracts cations while repelling anions, and thus cations are accumulated at the surface, while anion concentration is depleted.

Successively, we performed the calculation of the potential within the EDL at different Na^+^ concentrations and different distances from the electrolyte–graphene interface. In addition, graphene charge density and resistance for different Na^+^ concentrations were calculated using the Gouy–Chapman model as the first step, while in a second step, the Stern model was used for enhanced precision.

**Figure 3:**
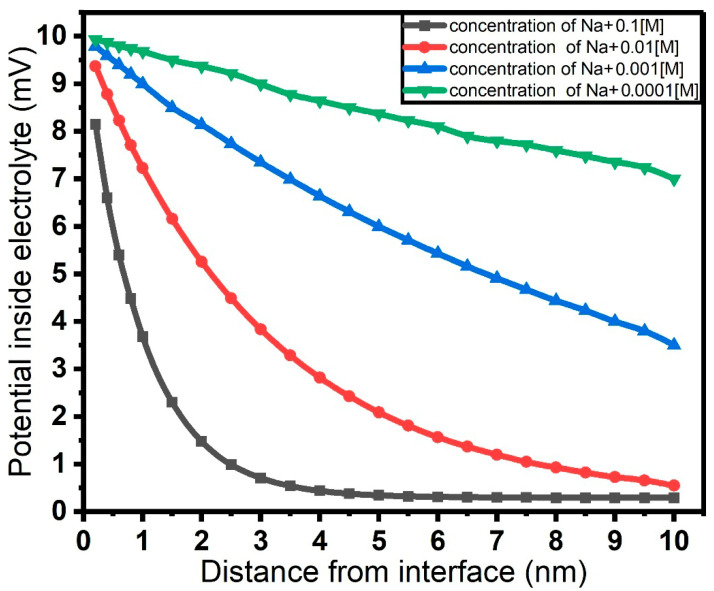
Electrical potential inside the electrolyte as a function of the distance from the electrolyte–graphene interface, for different concentrations of Na^+^. The computation uses the Stern model of the electric double layer (EDL).

Figure 3 reports the results of the calculations of potential as a function of distance from the interface. In this framework, increasing the concentration of Na^+^ ions near the graphene surface results in a shift in the Dirac point toward negative voltages, generated by electron accumulation in graphene, a compensatory mechanism to maintain charge neutrality upon Na^+^ ion accumulation. Pinning the graphene channel in our setup to a p-type behavior (positive Dirac point) in the absence of electrolyte gating, electron accumulation drives the system into an n-type characteristic. As indicated in Equation (8), the source–drain current depends linearly on the charge concentration in the graphene channel, which, in turn, might strongly depend on the presence of a specific analyte in the environment surrounding the channel: this sets a direct dependence of the channel electrical resistance on the analyte concentration.

**Figure 4:**
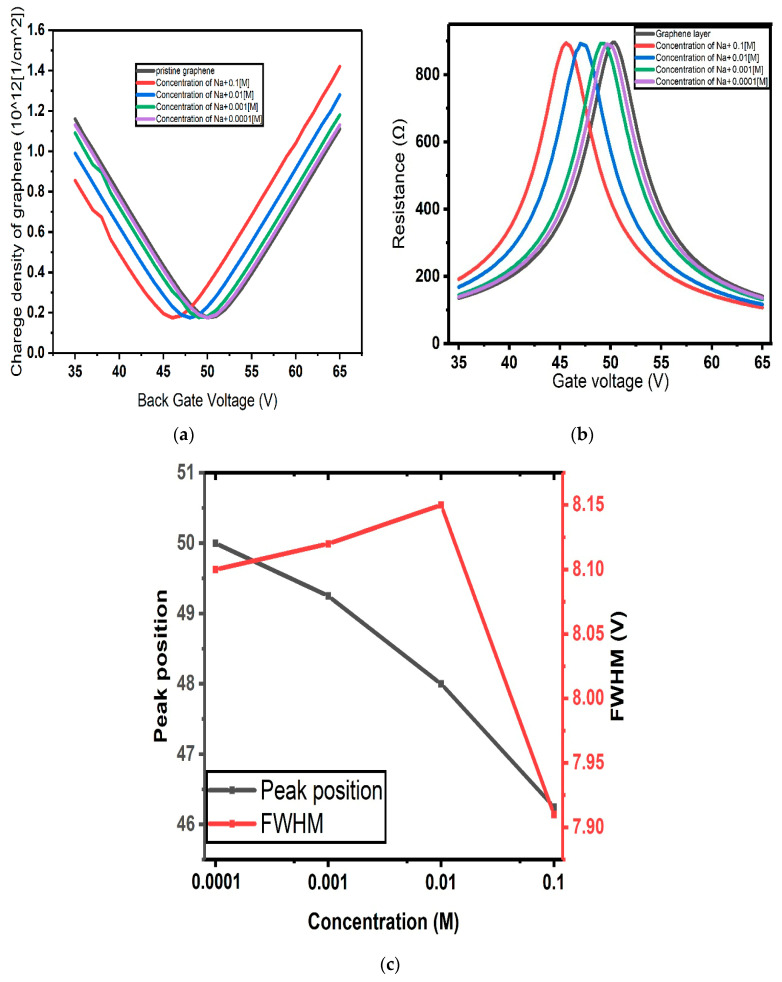
(**a**) Charge concentration and (**b**) resistance of graphene channel as functions of back gate voltage calculated according to the Gouy–Chapman model of EDL; (**c**) position of the resistance peak and its full width at half maximum (FWHM) as functions of the concentration.

The charge density and resistivity in the graphene channel as functions of the back gate voltage were computed upon different concentrations of Na^+^ ions in the range between 0.0 M (pristine graphene) and 0.1 M, spanning three orders of magnitude, and the results are reported in Figure 4a and Figure 4b, respectively. For each curve, peak position and full width at half maximum (FWHM) were extracted and plotted as functions of the analyte concentration, as shown in Figure 4c.

The capacitances in Figure 4 are calculated using the Gouy–Chapman model for the electrical double layer (EDL), where the differential capacitance depends on the Debye length, which varies with electrolyte concentration. This capacitance is used to compute the potential within the EDL, which determines the Dirac point shift in graphene based on Equation (12), with $C_{top}$ corresponding to the Gouy–Chapman capacitance. This approach quantifies the effect of ionic strength on device response.

**Figure 5:**
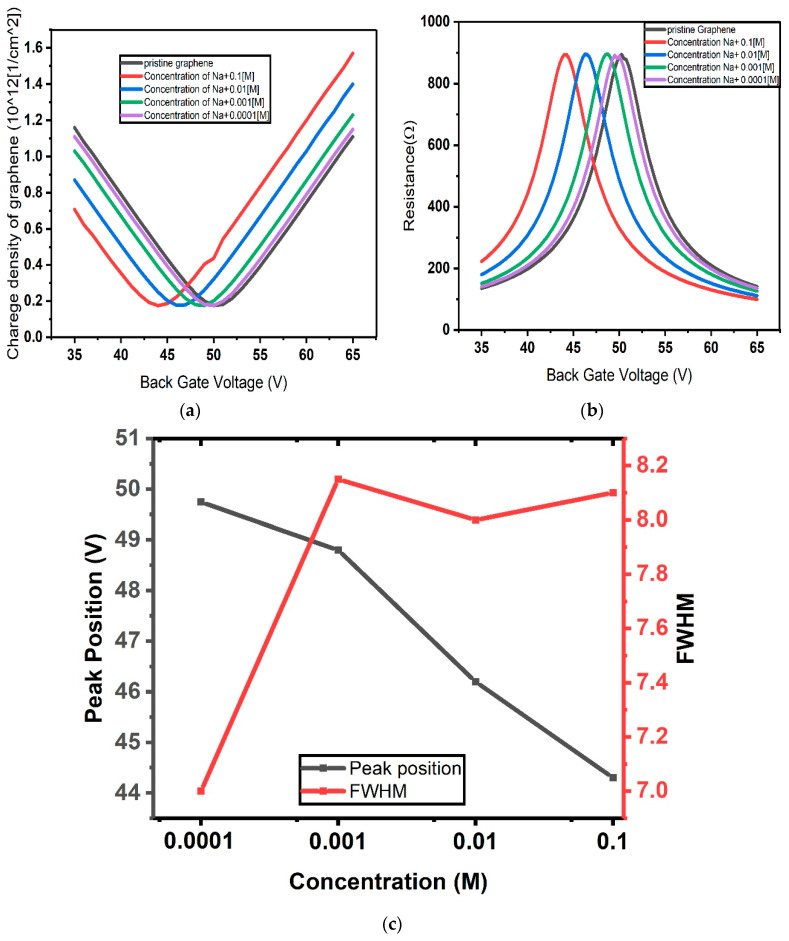
(**a**) Charge concentration and (**b**) resistance of graphene channel as functions of gate voltage calculated according to the Stern model of EDL; (**c**) position of the resistance peak and its full width at half maximum (FWHM) as functions of the concentration.

Figure 5 reports device charge density and electrical resistance versus back gate voltage for different Na^+^ concentrations, calculated using the Stern model, which accounts for both the capacitance of the diffusion layer—primarily influenced by the ion concentration—and of the Helmholtz layer—depending on the ionic radius. The incorporation of these additional parameters enhances the precision and accuracy of the calculations.

**Figure 6:**
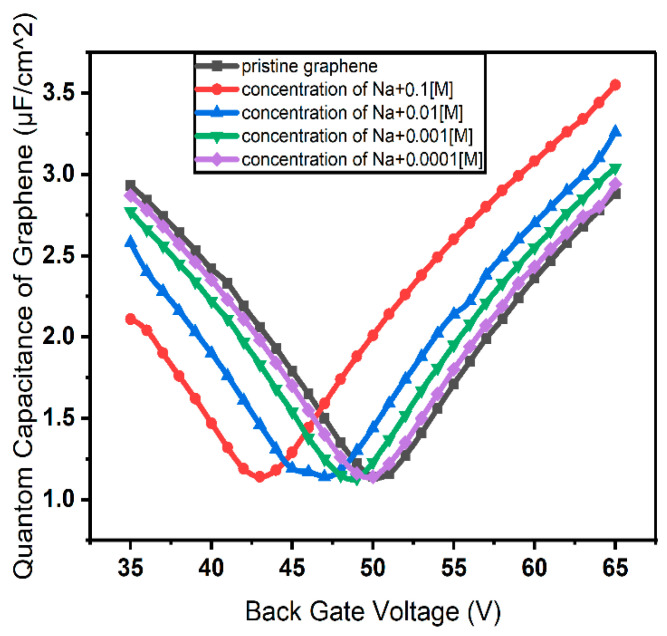
Quantum capacitance of graphene for different concentrations of Na^+^ versus back gate voltage.

**Figure 7:**
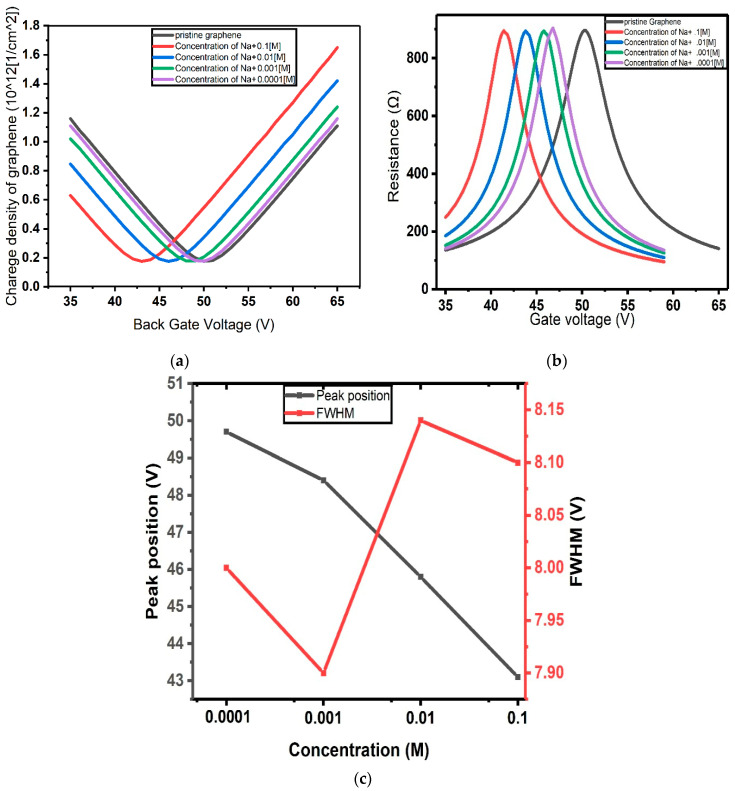
(**a**) Charge concentration and (**b**) resistance of graphene channel as functions of gate voltage, calculated using the Stern model of EDL by considering the impact of quantum capacitance of graphene; (**c**) position of the resistance peak and its full width at half maximum (FWHM) as functions of gate voltage.

In graphene field-effect transistors (GFETs) controlled by conventionally thick gate oxides, such as 300 nm thermal ${SiO}_{2}$, the total capacitance is predominantly governed by the capacitance of the gate oxide layer, while the quantum capacitance associated with the graphene channel can be neglected in first approximation. However, advanced device architectures feature the use of significantly thinner gate oxide layers with higher dielectric constants (κ), allowing a reduction in the device gate operating voltage and increasing the gate capacitance. In this frame, graphene quantum capacitance is expected to become very relevant; thus, extracting and analyzing it and rationalizing its onset and impact on transport properties becomes a crucial requirement. In this regard, the free-electron gas model can be assumed for the 2-dimensional electron system in graphene, with quantum capacitance expressed following [57]:

(14) $$C_{Q}=\frac{2e^{2}K_{B}T}{\pi ({\hbar v}_{f}{)}^{2}}Ln[2\left(1+cosh\frac{eV_{ch}}{K_{B}T}\right)]$$

where ħ is the Planck constant, e is the electron charge, $K_{B}$ is the Boltzmann constant, $v_{f}=\frac{C}{300}$ is the Fermi velocity of the Dirac electron (*c* represents the speed of light in a vacuum), and $V_{ch}=\frac{E_{f}}{e}$ is the potential of graphene. When $eV_{ch}\gg K_{B}T$, Equation (14) reduces to:

(15) $$C_{Q}=\frac{2e^{2}}{{\hbar v}_{f}\sqrt{\pi }}\sqrt{n}$$

where n is the carrier concentration [46]. In this frame, we calculated $\;C_{Q}$ associated with a pristine graphene channel, as well as with an electrolyte-gated graphene channel with different Na^+^ concentrations. The impact of the cation population on the Dirac point is reflected in the gate voltage corresponding to the minimum value that $C_{Q}$ assumes as a function of $V_{BG}$, as observed in Figure 6. The latter reports the $C_{Q}$ versus $V_{BG}$ curves computed for different values of the Na^+^ concentration, showing a marked shift in the curve minima towards lower voltages for increasing concentration. The impact of $C_{Q}$ on the device response can be estimated within the framework of the models discussed previously. As an example, the impact of $C_{Q}$ on device carrier density and resistance, for different concentrations of Na^+^ within the Stern model, is reported in Figure 7.

Importantly, the quantum capacitance is connected in series with the electric double layer (EDL) capacitance, $C_{EDL}$. Therefore, the top-gate capacitance $C_{top}$ can be calculated according to [9,53]:

(16) $$\frac{1}{C_{top}}=\frac{1}{C_{EDL}}+\frac{1}{C_{Q}}$$

Taking into account the quantum capacitance, as illustrated in Figure 7a and Figure 7b, enhanced sensor performance is expected, particularly in terms of sensitivity. In fact, the Dirac point shift in this case is approximately 7.5 V, compared to 6 V and 4.75 V for the Stern model and the Gouy–Chapman model, respectively. The GFET architecture discussed here is therefore well-suited for the detection of molecular systems at concentrations relevant to biological applications.

**Figure 8:**
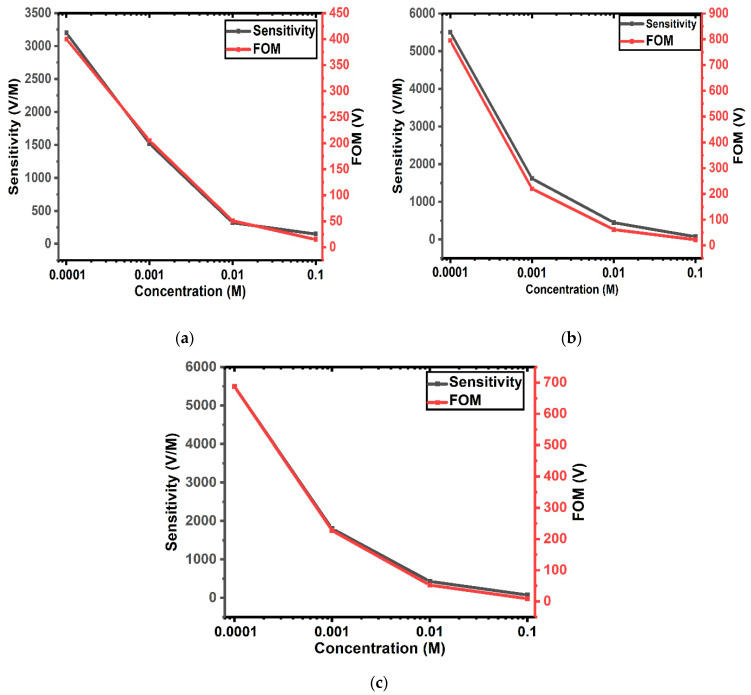
Sensor sensitivity and FOM as functions of analyte concentration: (**a**) Gouy–Chapman model of EDL; (**b**) Stern model of the EDL; and (**c**) Stern model of EDL including the effect of graphene quantum capacitance.

Within this context, the performance of our devices can be quantified in terms of the sensor sensitivity, *S*, and figure of merit, FOM. The first is defined as the slope of the linear region of the device response curve [7,58]:

(17) $$S=\frac{\Delta X}{\Delta N}$$

where ΔX is the change in the electrical signal and ΔN  is the corresponding change in concentration. FOM is defined as [58]:

(18) $$FOM=\frac{S}{FWHM}$$

with FWHM as full width at half maximum. As illustrated in Figure 8a–c, sensitivity values up to 3200 V/M, 5500 V/M, and 5620 V/M are envisioned for the Gouy–Chapman model, the Stern model, and the model incorporating quantum capacitance effects, respectively. The GFET biosensor parameters computed for the Gouy–Chapman model, the Stern model, and the model incorporating quantum capacitance effects are presented in Table 1, Table 2 and Table 3.

## 4. Conclusions

We have investigated the correlation between different models of electric double layers in sodium ion-based electrolytes, enabling the gating of graphene field-effect transistors and discussing the potential for high-sensitivity detection of Na^+^ concentration and, virtually, of any charged molecular species in the electrolyte. Quantum capacitance of graphene is taken into account, providing higher sensitivity compared to the Gouy–Chapman and Stern models. We computed how the resistance and the charge carrier density in the graphene channel at the Dirac point shift from higher to lower potential, as functions of the Na^+^ concentration at the graphene surface and of the used EDL model. On the one hand, these findings provide a computational framework for improving accuracy in device response prediction based on the incorporation of realistic EDL models. On the other hand, the approach can be naturally extended to virtually any type of ions, with each ion bringing its specific ionic radius, which, in turn, determines the Helmholtz capacitance and the Debye length, defining the Gouy–Chapman capacitance; in other words, the Dirac point shift can be taken as the fingerprint of a specific ion type. The GFET architectures discussed here are therefore ideally suited for detecting molecular systems at concentrations relevant to biological applications, enabling the detection not only of ions but also of proteins. This highlights their potential as versatile biosensors for a wide range of analytes.

## Figures and Tables

**Table 1:** GFET biosensor parameters computed for the Gouy–Chapman model of electric double layer.

Concentration	FWHM (V)	Sensitivity (V/M)	FOM (V)
0.0001 [M]	8.1	3200	400
0.001 [M]	8.12	1520	205
0.01 [M]	8.15	320	51
0.1 [M]	7.9	150	15

**Table 2:** GFET biosensor parameters computed for the Stern model of electric double layer without considering quantum capacitance.

Concentration	FWHM (V)	Sensitivity (V/M)	FOM (V)
0.0001 [M]	7	5500	795
0.001 [M]	8.19	1620	220
0.01 [M]	8.1	450	62
0.1 [M]	8.15	75	22

**Table 3:** GFET biosensor parameters computed for the Stern model of electric double layer incorporating quantum capacitance.

Concentration	FWHM (V)	Sensitivity (V/M)	FOM (V)
0.0001 [M]	7.97	5620	700
0.001 [M]	7.9	1780	215
0.01 [M]	8.13	490	59
0.1 [M]	8.1	320	12

## Data Availability

The original contributions presented in this study are included in the article. Further inquiries can be directed to the corresponding author.

## Supplementary Materials

The following supporting information can be downloaded at: https://www.mdpi.com/article/10.3390/mi17040433/s1, Figure S1: Meshing features employed in the simulations, with different level of refinement.
