# Supplementary material for: Graphene-Based Chemical Field-Effect Transistors: Impact of Electric Double Layer Model and Quantum Capacitance on Na+ Detection Capabilities
Source: Micromachines (Basel). 2026 Mar 31;17(4):433. doi: 10.3390/mi17040433 (PMC13117873; doi:10.3390/mi17040433)
Supplement: Supplementary file 1 [file micromachines-17-00433-s001.zip › micromachines-4089015-supplementary.pdf]

Graphene-Based Chemical Field-Effect Transistors: Impact of Electric Double Layer Model and Quantum Capacitance on Na^+ Detection Capabilities

Ghassem Baridi [1,*], Arsalan Liaquat [2], Leonardo Martini [2], Luca Nappi [2], Federico Rapuzzi [2], Vito Clericò [3], El Hadj Abidi [3], Yahya Moubarak Meziani [3], Mario Amado [3], Enrique Diez [3], Giorgia Brancolini [4], Luigi Rovati [1] and Francesco Rossella [2]

[1] Department of Engineering "Enzo Ferrari", University of Modena and Reggio Emilia, Via P. Vivarelli, 10, 41125 Modena, Italy
[2] Department of Physics, Computer Science and Mathematics, University of Modena e Reggio Emilia, Via Campi 213/a, 41125 Modena, Italy
[3] Department of Applied Physics, University of Salamanca, 37008 Salamanca, Spain
[4] Istituto Nanoscienze—CNR, S3, Via G. Campi 213/A, 41125 Modena, Italy
* Correspondence: ghassem.baridi@unimore.it

SI.1 Preliminary molecular dynamics study.

It is worth mentioning that GFET architectures similar to the one discussed in the present work are well suited for the detection of molecular systems at concentrations relevant to biological applications, and the entire system can be quantitatively investigated resorting on molecular dynamics. In this frame, we have tackled the interaction of wild-type and D76N beta2-microglobulin (β2-m) on graphite, a representative hydrophobic surface, using straight MD and Temperature Replica Exchange MD (T-REMD), following the methodology described in the PhD thesis of Maschio [47]. Here, "wilde type" refer to the naturally occurring form of β2-m, while "D76N" denotes a specific mutant variant in which the aspartic acid at position 76 is replaced by asparagin. The results of the simulations, although quite preliminary and still under refinement, seem to indicate fast adsorption, accompanied by partial unfolding, particularly for the D76N variant. Perspectives of integration of the molecular dynamics approach with the computational strategies developed in the present work are currently under investigation.

SI 2.a Introduction to the model used in this work including convergence of calculations versus mesh size.

The COMSOL Multiphysics model considers a pH-7 aqueous droplet placed on the graphene surface, where the formation of an electric double layer at the graphene–electrolyte interface governs the electrostatic potential distribution and the resulting Dirac point shift. Graphene is modeled as a charged boundary interacting with mobile ions in the electrolyte, while bulk boundary

conditions are applied far from the interface to ensure potential decay. Due to the large disparity in length scales between the graphene layer and the surrounding domains, a non-uniform meshing strategy is adopted, with strong refinement near the graphene–electrolyte interface and coarser elements elsewhere. Mesh-convergence tests confirm that the calculated potential profiles and Dirac point shifts are insensitive to further mesh refinement. Figure S1 shows the mesh distribution used in the simulations.

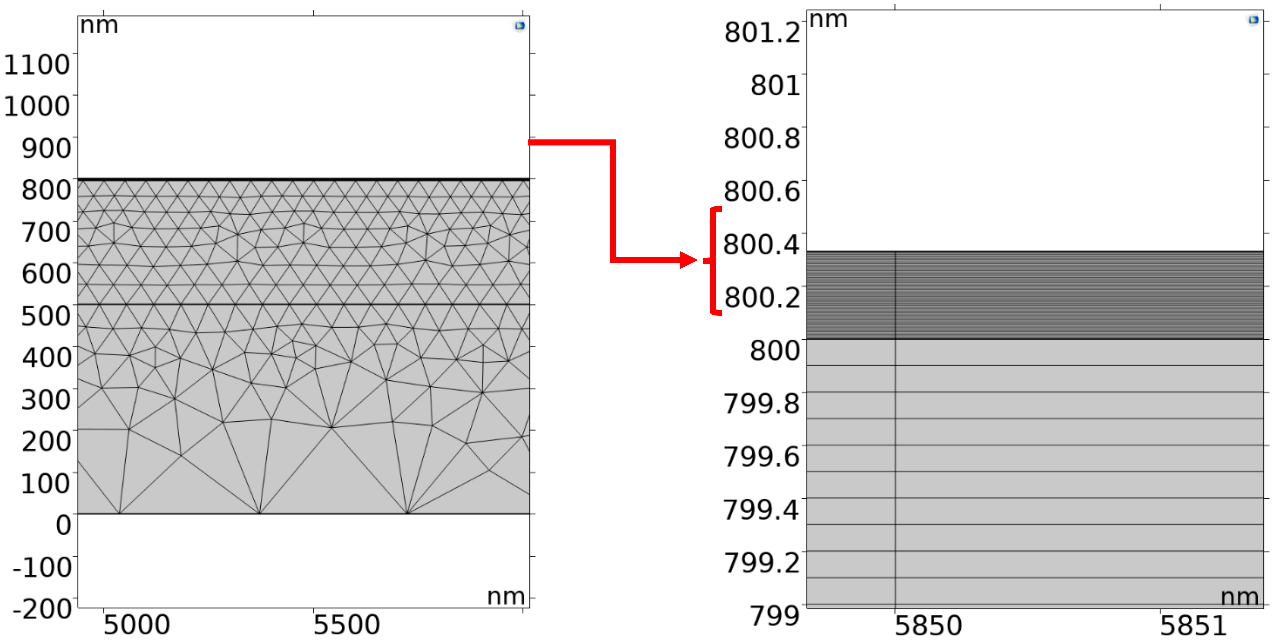

Figure S1. Meshing features employed in the simulations, with different level of refinement. (left) overall view of the computational mesh for the graphene field-effect transistor; (right) zoomed-in view highlighting the finer mesh applied in the graphene layer and the adjacent region to improve numerical accuracy.

SI.2. b. Model details

The Helmholtz model describes the relation between the electrical potential and space charge distributions with the one-dimensional Poisson equation [38,39,40,41]:

$$\frac{d^2\varphi}{dx^2} = -\frac{\rho(x)}{\varepsilon_r\varepsilon_0} \qquad \text{(S1)},$$

where φ represents the electric potential, ρ is the charge density, x denotes the distance from the electrode, ε_0 is the vacuum permittivity, and ε_r is the relative permittivity of the surrounding medium. This approach models the ions as point charges, which enables the reformulation of Equation (S1) to describe the region between the two layers [39,40,41]:

$$\frac{d^2\varphi}{dx^2} = 0 \quad \text{(S2)}$$

The Helmholtz layer can be modelled as a parallel-plate capacitor, with its capacitance expressed as [34,40,41]:

$$C_H = \frac{\varepsilon_r \varepsilon_0}{l} \quad \text{(S3)}$$

where $l = \frac{d}{2}$, d being the distance corresponding to the radius of attracted of the ion to the electrode surface. The model does not account for the dependence of the measured capacitance on electrolyte concentration and does not consider the thermal motion of ions in the solution.

Differently, the Gouy and Chapman models consider how ions behave thermally near a charged interface, introducing the concept of a Diffuse Double Layer (DDL), where oppositely charged ions (counterions) are drawn toward the surface, while similarly charged ions (co-ions) are pushed away. These distributions occur within a dielectric medium and are described by the Boltzmann equation [39,40]:

$$c_i = c_{\pm}^{\infty} \exp\left(\frac{-Z_{\pm} e\varphi}{K_B T}\right) \quad \text{(S4)}$$

where $c_+$and c_- represent the local concentrations of cations and anions, respectively, while $c_{\pm}^{\infty}$ denotes their concentrations in the bulk solution. $Z_{\pm}$ is the valence (charge number) of the ions, e is the elementary charge, K_B is the Boltzmann constant, and T is the absolute temperature. The total charge density per unit volume including all ionic species is obtained by summing the contributions from each individual ion:

$$\rho(x) = \sum_i c_{\pm} Z_{\pm} e = \sum_i e Z_{\pm} c_{\pm}^{\infty} \exp\left(\frac{-Z_{\pm} e\varphi}{K_B T}\right) \quad \text{(S5)}$$

Combining Eq. (S1) and Eq. (S5) leads to the Poisson-Boltzmann equation:

$$\frac{d^2\varphi}{dx^2} = -\frac{e}{\varepsilon_r \varepsilon_0} \sum_i Z_{\pm} c_{\pm}^{\infty} \exp\left(\frac{-Z_{\pm} e\varphi}{K_B T}\right) \quad \text{(S6)}$$

if $K_B T >> |ze\varphi|$, then the exponent term in Equation (S6) can be as:

$$\frac{d^2\varphi}{dx^2} = -\sum \frac{Z_{\pm} c_{\pm}^{\infty} e}{\varepsilon_r \varepsilon_0} + \frac{e}{\varepsilon_r \varepsilon_0} \sum_i Z_{\pm} n_{\pm}^{\infty} \left(\frac{-Z_{\pm} e\varphi}{K_B T}\right) \quad \text{(S7)}$$

The first term on the right-hand side of Equation (S7) is zero, as it pertains to the total charge of the electrolyte in the bulk. So, the potential reads:

$$\varphi = \varphi_0 \exp\left(\frac{xe^2}{\varepsilon_r \varepsilon_0 K_B T}\right) \sum Z_{\pm}^2 c_{\pm}^{\infty} \quad \text{(S8)}$$

The expression $I = \mu = 1/2 \sum c_i^{\pm} Z^2$ quantifies the impact of charge and interionic interactions on the electrolyte feature referred to as bulk ionic strength [40,41]. The characteristic thickness of the ionic cloud surrounding a central ion can be expressed within the Debye–Hückel theory as the Debye length, and reads [42]:

$$\lambda_D = \sqrt{\frac{\varepsilon_r \varepsilon_0 T K_B}{e^2 \sum Z_{\pm}^2 c_{\pm}^{\infty}}} \qquad \text{(S9)}$$

If it is substituted in (S8), Equation (S9) is obtained:

$$\varphi(x) = \varphi_0(x) \exp\left(\frac{-x}{\lambda_D}\right) \qquad \text{(S10)}$$

For a symmetrical (Z_Z) electrolyte, Eq. (S10) has the below form [37,39,40]:

$$\frac{\partial \varphi}{\partial x} = \left(\frac{8 K_B T c_{\pm}^{\infty}}{\varepsilon_r \varepsilon_0}\right)^{\frac{1}{2}} \sinh\left(\frac{Z_{\pm} e \varphi}{2 K_B T}\right) \qquad \text{(S11)}$$

The charge density of the diffuse layer is [43,44,45]:

$$\sigma_M = \varepsilon_r \varepsilon_0 \left(\frac{d\varphi}{dx}\right)_{x=0} = (8 K T c_{\pm}^{\infty} \varepsilon_r \varepsilon_0)^{\frac{1}{2}} \sinh\left(\frac{Z_{\pm} e \varphi}{2 K_B T}\right) \qquad \text{(S12)}$$

By differentiating, the differential capacitance is obtained as [46,47,48]:

$$C_{GC} = \frac{d\varphi_M}{d\varphi_0} = \left(\frac{2 e^2 c_{\pm}^{\infty} \varepsilon_r \varepsilon_0 Z^2}{K_B T}\right)^{\frac{1}{2}} \cosh\left(\frac{2 e \varphi}{2 K T}\right) \qquad \text{(S13)}$$

By replacing equation number (S9) in equation number (S13) the capacitance in Gouy-Chapman it will be in below form.

$$C_{Gc} = \frac{\varepsilon_r \varepsilon_0}{\lambda_D} \cosh\left(\frac{2 e \varphi}{2 K_B T}\right) \qquad \text{(S14)}$$

In 1924, Stern enhanced the understanding of the EDL theory by introducing a more accurate representation of the interfacial structure. He merged two earlier models and compute capacitance of EDL. Mathematically, the differential capacitance of the electrical double layer, denoted as C_{EDL}, can be described as two capacitors connected in series:

$$\frac{1}{C_{EDL}} = \frac{1}{C_H} + \frac{1}{C_{Gc}} \qquad \text{(S15)}$$

where C_H is the capacitance of the charges held inside the Helmholtz layer and C_{Gc} is the capacitance of the diffuse layer [49].
